# A novel nomogram for predicting the decision to delayed extubation after thoracoscopic lung cancer surgery

**DOI:** 10.1080/07853890.2022.2160490

**Published:** 2023-03-04

**Authors:** Chaoyang Tong, Qing Miao, Jijian Zheng, Jingxiang Wu

**Affiliations:** a Department of Anesthesiology, Shanghai Chest Hospital, Shanghai Jiao Tong University, Shanghai, China; b Department of Anesthesiology, Shanghai Children’s Medical Center, School of Medicine, Shanghai Jiao Tong University, Shanghai, China

**Keywords:** Thoracoscopic surgery, lung cancer, delayed extubation, nomogram

## Abstract

**Objective:**

Delayed extubation was commonly associated with increased adverse outcomes. This study aimed to explore the incidence and predictors and to construct a nomogram for delayed extubation after thoracoscopic lung cancer surgery.

**Methods:**

We reviewed medical records of 8716 consecutive patients undergoing this surgical treatment from January 2016 to December 2017. Using potential predictors to develop a nomogram and using a bootstrap-resampling approach to conduct internal validation. For external validation, we additionally pooled 3676 consecutive patients who underwent this procedure between January 2018 and June 2018. Extubation performed outside the operating room was defined as delayed extubation.

**Results:**

The rate of delayed extubation was 1.60%. Multivariate analysis identified age, BMI, FEV_1_/FVC, lymph nodes calcification, thoracic paravertebral blockade (TPVB) usage, intraoperative transfusion, operative time and operation later than 6 p.m. as independent predictors for delayed extubation. Using these eight candidates to develop a nomogram, with a concordance statistic (C-statistic) value of 0.798 and good calibration. After internal validation, similarly good calibration and discrimination (C-statistic, 0.789; 95%CI, 0.748 to 0.830) were observed. The decision curve analysis (DCA) indicated the positive net benefit with the threshold risk range of 0 to 30%. Goodness-of-fit test and discrimination in the external validation were 0.113 and 0.785, respectively.

**Conclusion:**

The proposed nomogram can reliably identify patients at high risk for the decision to delayed extubation after thoracoscopic lung cancer surgery. Optimizing four modifiable factors including BMI, FEV_1_/FVC, TPVB usage, and operation later than 6 p.m. may reduce the risk of delayed extubation.

**Key Messages:**
- This study identified eight independent predictors for delayed extubation, among which lymph node calcification and anaesthesia type were not commonly reported.
- Using these eight candidates to develop a nomogram, we could reliably identify high-risk patients for the decision to delayed extubation.
- Optimizing four modifiable factors, including BMI, FEV_1_/FVC, TPVB usage, and operation later than 6 p.m. may reduce the risk of delayed extubation.

## Introduction

Tracheal extubation after surgery is a critical step in a patient’s recovery from general anaesthesia (GA). And more than 20% of major complications associated with airway management occur around the time of extubation, with severe consequences such as hypoxemia and death [1,2]. Although many guidelines focus on endotracheal intubation, the literature on tracheal extubation at the end of operation is limited [3,4]. Prolonged endotracheal intubation is not a beneficial process, which may result in related airway complications regarding pneumonia, laryngeal injury, glottic stenosis and dysphagia [5–8]. Also, it takes up the limited medical resource, greatly reducing the transition efficiency in post-anaesthesia care unit (PACU) and intensive care unit (ICU) [9]. Thus, it is of great clinical significance to identify high-risk patients and adjust predictors of delayed extubation in order to reduce its incidence and related complications.

With the ageing of the population and the promotion of low-dose computed tomography (LDCT) screening for lung cancer [10], there will be an increasing number of patients and accompanied with severity of comorbidities in thoracic surgery. In addition, the specific attributes of thoracic surgery, such as unique physiology of one-lung ventilation (OLV) including hypoperfusion or reperfusion, atelectasis or reinflation, and increased inflammatory mediators and postoperative incisional chest wall pain, all increase the uncertainty of postoperative extubation [11,12]. Currently, underlying predictors for delayed extubation have been explored in several fields such as cardiac, thoracotomy, neurosurgical, liver transplantation and spine populations [9,13–17]. However, to date, no document has researched predictors for delayed extubation after thoracoscopic lung cancer surgery. Besides, many of the published factors associated with delayed extubation cannot be modified [18]. Further, no effective and well-performing model was established for predicting the possibility of delayed extubation.

Therefore, by reviewing a large number of medical records, this study aims to explore the incidence and potential predictors and to construct a nomogram to predict the decision to delayed extubation after this surgery. Moreover, considering the reported influence of timing of operation and attending handoff on delayed extubation in other surgical specialties [9,18,19], this study also attempts to investigate the interaction of the two factors on delayed extubation.

## Materials and methods

### Ethics

This single-centre retrospective study was performed following the approval of the Institutional Review Board (IRB) of Shanghai Chest Hospital (Chair: Dr. Zheng Ning, permission NO.IS22039) on 7 June 2022, with informed consent waived. This article adheres to STROBE guidelines [20].

### Study design and patients

Between January 2016 to December 2017, we reviewed medical records of 8716 consecutive patients undergoing thoracoscopic lung cancer surgery to identify predictors and to construct a nomogram for the decision to delayed extubation. For the external validation of the proposed model, we additionally pooled 3676 consecutive patients who underwent this surgical treatment between January 2018 and June 2018. Excluded patients were described in the flow diagram (Supplementary Figure 1).

### Data collection, outcomes and definition

Perioperative clinical data were prospectively extracted from our institution’s electronic medical records, including patient’s preoperative characteristics and intraoperative variables such as tumour size, advanced tumour stage (*T* ≥ 2), hypoxemia, lymph nodes calcification and involvement, type of resection, approach, anaesthesia type, location of resection and so on. Hypoxemia was defined as SPO_2_≤90% lasting for 5 min. Based on previously published document, 6 p.m. was used as the watershed for the timing of the operation, considering that the onset of thoracoscopic lung surgery later than 6 p.m. developed an increased risk of intraoperative complications [21]. Extubation performed outside the operating room was defined as delayed extubation, which was determined by the anaesthesiologist and thoracic chief surgeon in view of the patient’s preoperative status and intraoperative adverse events [5,9,15,19].

### Statistical analysis

Statistical power calculations were not performed prior to this study since the sample size was based on available data in our dataset. Statistics and data analysis plans were defined before accessing the data and were completed after the data were accessed. Continuous variables were compared using Two independent sample t-test or Mann-Whitney U test based on the occurrence of delayed extubation. Categorical variables were compared with Chi-square test or Fisher exact test, depending on the sample size. Univariate analysis showed that all factors significantly correlated with delayed extubation (*p* < .2) were inserted into the multivariate logistic regression model using the forward selection strategy.

The predictive model was presented with a nomogram to provide a visual point system to estimate the probability of delayed extubation. Hosmer-Lemeshow (H-L) goodness-of-fit test was used to evaluate the model’s fit. Discrimination (concordance statistic, C-statistic) and calibration (calibration curves) were used to evaluate the performance of the prediction model. To reduce overfitting and quantify optimism, the nomogram was internally validated with an approach to 1000 bootstrapped resampling and calculating an optimism-corrected C-statistic. Decision curve analysis (DCA) was used to assess the clinical validity and net benefit of the nomogram [22].

For external calibration, the differences between the mean predicted and observed probability were compared by using a H-L type χ^2^ statistic. And a large *p* value (>.05) indicates good calibration. The area under the receiver operating characteristic curve (AUROC) and the optimal cutoff values were calculated to assess model’s discrimination. Statistical analysis was performed using the SPSS 26.0 software (IBM Corp., Armonk, NY, USA). R version 4.1.2 was used with the packages of rms, rmda, forestplot, tidyr, dplyr, pROC, ResourceSelection and PredictABEL. *p* Value < .05 was considered statistically significant.

## Results

### Study cohort

From January 2016 to June 2018, 12,392 patients underwent thoracoscopic lung cancer surgery, of which 21.3% (2645 out of 12,392) underwent segmentectomy resection and 78.7% (9747 out of 12,392) underwent lobectomy resection, 1.60% (139 out of 8716) and 1.40% (50 out of 3676) occurred delayed extubation in the development and validation cohorts. The rate of thoracic paravertebral blockade (TPVB) usage in the development and validation cohorts was 11.2% and 22.8%, respectively.

### Model development

**Figure 1.**
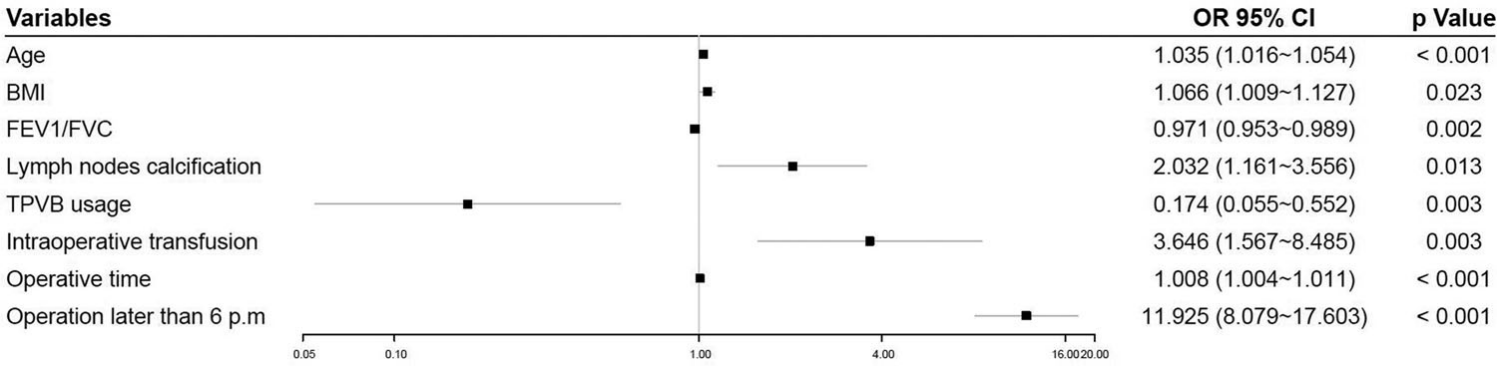
Forest plot of independent predictors of delayed extubation. BMI: body mass index; FEV_1_: forced expiratory volume in 1 s; FVC: forced vital capacity; TPVB: thoracic paravertebral blockade.

**Figure 2.**
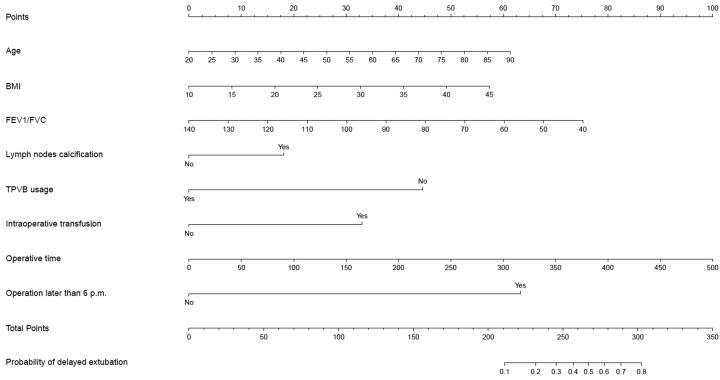
A novel nomogram to predict delayed extubation. The nomogram provides a visual point system based on the combination of patient characteristics (age, BMI, FEV_1_/FVC, lymph nodes calcification, combined with TPVB, intraoperative transfusion, operative time and operation later than 6 p.m.) to estimate the probability of delayed extubation. To calculate the probability of delayed extubation, the points of eight variables determined on the scale were added to obtain the total points. Draw a vertical line from the total points scale to the last axis to obtain the corresponding probability of delayed extubation. BMI: body mass index; FEV_1_: forced expiratory volume in 1 s; FVC: forced vital capacity; TPVB: thoracic paravertebral blockade.

Univariate analysis identified that fifteen variables were significantly associated with delayed extubation in the development cohort (Table 1). Multivariate analysis identified that older age (Odds ratio (OR)=1.035, 95%CI, 1.016 to 1.054, *p* < .001), elevated BMI (OR = 1.066, 95%CI, 1.009 to 1.127, *p* = .023), increased FEV_1_/FVC (OR = 0.971, 95%CI, 0.953 to 0.989, *p* = .002), lymph nodes calcification (OR = 2.032, 95%CI, 1.161 to 3.556, *p* = .013), GA plus TPVB (vs GA alone, OR = 0.174, 95%CI, 0.055 to 0.552, *p* = .003), intraoperative transfusion (OR = 3.646, 95%CI, 1.567 to 8.485, *p* = .003), prolonged operative time (OR = 1.008, 95%CI, 1.004 to 1.011, *p* < .001), and operation later than 6 p.m. (OR = 11.925, 95%CI, 8.079 to 17.603, *p* < .001) were independent predictors for delayed extubation (Figure 1). Using these eight candidates to develop a nomogram to predict the probability of delayed extubation (Figure 2).

**Table 1.** Preoperative and intraoperative patient characteristics.

	Development cohort (*n* = 8716)			Validation cohort (*n* = 3676)		
Variables^a^	Delayed extubation (*n* = 139)	In-OR extubation (*n* = 8577)	*p* Value	Delayed extubation (*n* = 50)	In-OR extubation (*n* = 3626)	*p* Value
Age, years	62.2 ± 10.0	57.8 ± 10.6	<.001^b^	60.9 ± 11.1	58.1 ± 10.7	.067
Sex			.002^b^			.127
Male sex	73 (52.5)	3391 (39.5)		25 (50.0)	2198 (60.6)	
Female sex	66 (47.5)	5186 (60.5)		25 (50.0)	1428 (39.4)	
BMI, kg/m^2^	24.0 ± 3.5	23.2 ± 3.0	.013^b^	24.0 ± 3.0	23.3 ± 3.0	.093
ASA grade			.267			.545
I	8 (5.8)	808 (9.4)		2 (4.0)	291 (8.0)	
II	114 (82.0)	6906 (80.5)		44 (88.0)	2997 (82.7)	
III/IV	17 (12.2)	863 (10.1)		4 (8.0)	338 (9.3)	
FEV_1_/FVC, %	98.7 ± 11.1	101.2 ± 7.5	.008b	101.7 ± 10.1	101.2 ± 8.3	.677
DLCO%	90.7 ± 13.7	94.5 ± 15.0	.003b	92.4 ± 17.0	94.5 ± 16.5	.379
Comorbidity						
Hypertension	12 (8.6)	565 (6.6)	.336	5 (10.0)	290 (8.0)	.795
Diabetes mellitus	5 (3.6)	277 (3.2)	.806	3 (6.0)	131 (3.6)	.428
Coronary artery disease	1 (0.7)	37 (0.4)	1.000	2 (2.6)	56 (1.1)	.199
Stroke/TIA	0 (0)	17 (0.2)	1.000	0 (0)	17 (0.5)	1.000
Chemoradiotherapy	1 (0.7)	30 (0.3)	.393	2 (4.0)	4 (0.1)	.003b
Tumour size, cm	2.1 ± 1.4	1.7 ± 1.1	.003^b^	2.1 ± 1.1	1.7 ± 1.0	.016b
Advanced T stage (T ≥ 2)	23 (16.5)	901 (10.5)	.022^b^	11 (22.0)	319 (8.8)	.004b
Hypoxemia	7 (5.0)	347 (4.0)	.557	5 (10.0)	111 (3.1)	.020b
Lymph nodes calcification	17 (12.2)	391 (4.6)	<.001^b^	5 (10.0)	177 (4.9)	.099
Clinical nodal involvement	13 (9.4)	426 (5.0)	.019^b^	4 (8.0)	169 (4.7)	.296
Pleural adhesions	3 (2.2)	198 (2.3)	1.000	3 (6.0)	67 (1.8)	.069
Type of resection			.724			.860
Segmentectomy resection	27 (19.4)	1771 (20.6)		11 (22.0)	836 (23.1)	
Lobectomy resection	112 (80.6)	6806 (79.4)		39 (78.0)	2790 (76.9)	
Approach			.183			1.000
VATS	136 (97.8)	8190 (95.5)		48 (96.0)	3498 (96.5)	
RATS	3 (2.2)	387 (4.5)		2 (4.0)	128 (3.5)	
Anaesthesia type			.001^b^			.066
GA alone	136 (97.8)	7606 (88.7)		44 (88.0)	2793 (77.0)	
Combined with TPVB	3 (2.2)	971 (11.3)		6 (12.0)	833 (23.0)	
Location of resection			.530			.819
Left resection	57 (41.0)	3293 (38.4)		20 (40.0)	1393 (38.4)	
Right resection	82 (59.0)	5284 (61.6)		30 (60.0)	2233 (61.6)	
Conversion to thoracotomy	9 (6.5)	113 (1.3)	<.001^b^	2 (4.0)	33 (0.9)	.081
Intraoperative arrhythmia	3 (2.2)	146 (1.7)	.734	2 (4.0)	49 (1.4)	.152
Intraoperative transfusion	8 (5.8)	81 (0.9)	<.001^b^	4 (8.0)	33 (0.9)	.001^b^
Operative time, mins	117.0 ± 49.7	97.2 ± 36.4	<.001^b^	124.7 ± 67.2	96.4 ± 35.8	.001^b^
Attending handoff	9 (6.5)	107 (1.2)	<.001^b^	7 (14%)	54 (1.5)	<.001^b^
Operation later than 6 p.m.	44 (31.7)	345 (4.0)	<.001^b^	22 (44.0)	134 (3.7)	<.001^b^

^a^Continuous data are shown as mean ± standard deviation and categoric data as number (%).

^b^Statistically significant (P<0.05).

*Note:* OR: operating room; BMI: body mass index; ASA: American Society of Anaesthesiology; FEV_1_: forced expiratory volume in 1 s; FVC: forced vital capacity; DLCO: diffusion capacity for carbon monoxide; TIA: transient cerebral ischemic attack; VATS: video-assisted thoracoscopic surgery; RATS: robotic-assisted thoracoscopic surgery; GA: general anaesthesia; TPVB: thoracic paravertebral blockade.

### Model performance and internal validation

**Figure 3.**
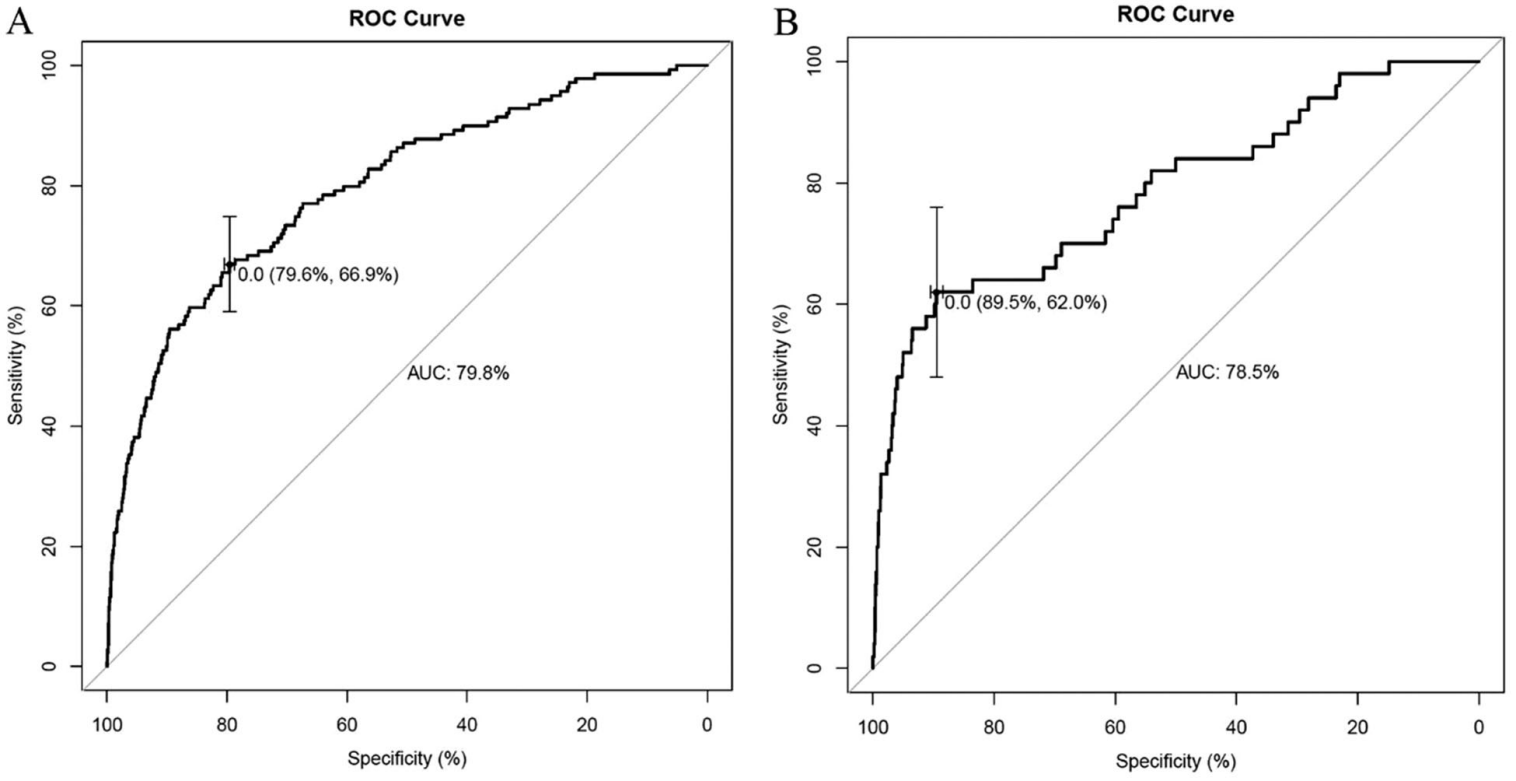
AUROC curve analysis of the development (A) and validation cohort (B).

**Figure 4.**
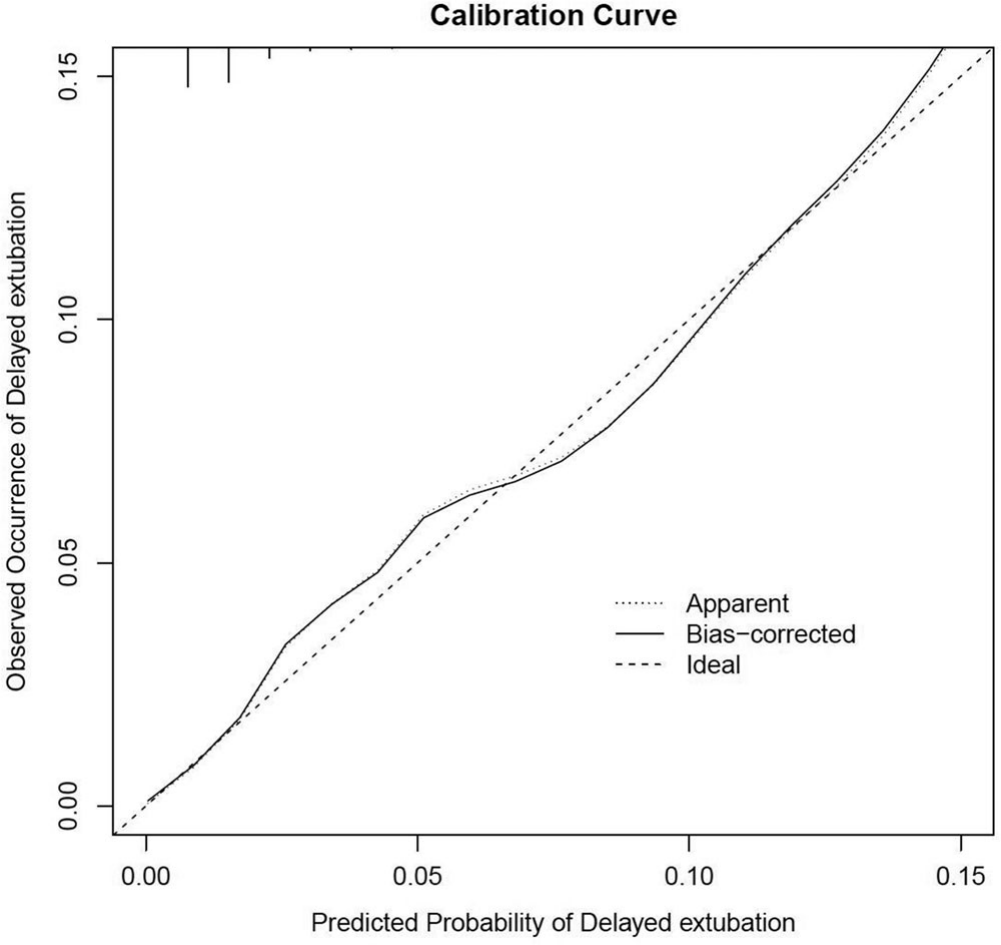
Internal calibration curves. A completely accurate prediction model will generate a plot where the probability of the actual observed and predicted corresponding completely and fall along the 45°line (dashed line). The apparent calibration curve (dotted line) represents the calibration of the model in the development data set, while the bias-corrected curve (solid line) is the calibration result after correcting the optimism with the 1000 bootstrap-resampling.

H-L goodness-of-fit test value was 0.527. The C-statistic value of the prediction model was 0.798 (95%CI, 0.758 to 0.838), which showed good discrimination. The sensitivity and specificity based on AUROC curve were 66.9% and 79.6%, respectively (Figure 3(A)). The apparent calibration curve was close to the 45° ideal line, indicating that the observed probability was consistent with predicted probability in the development cohort (Figure 4). To reduce the optimism of the model, internal validation with 1000 bootstrap approach was conducted, which reflect good discrimination with optimism-corrected C-statistic of 0.789 (95%CI, 0.748 to 0.830). And the bias-corrected calibration curve also demonstrated that the prediction model was well calibrated when the actual observed probability of delayed extubation was less than 15% (Figure 4).

### DCA for the development prediction model

**Figure 5.**
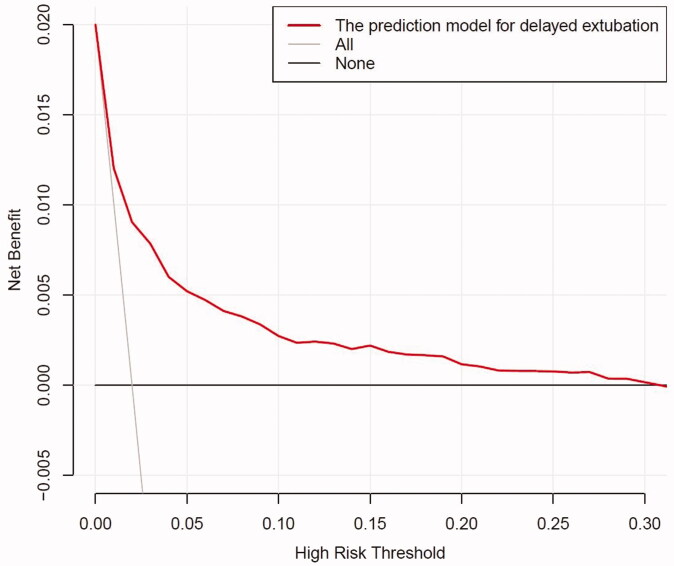
The DCA shows the clinical usefulness of the nomogram. The Y-axis represents net benefit. The bold solid black line is a nomogram predicting the risk of delayed extubation. The solid grey line indicates that all patients occurred delayed extubation, while the fine solid black line indicates that no patient occurred delayed extubation. This DCA could provide a larger net benefit, with ranges of 0% to 30%. DCA: decision curve analysis .

The depicted DCA was used to determine whether decisions based on the predictive model had clinical applicability compared to the default strategy. Such analyses provide insight into the range of predicted risk for which the model has a high net benefit than simply either treating all (slope line) patients versus treating no (horizontal line) patient, that is to say, a prediction model is only useful at the threshold risk. The graphically DCA indicated the expected net benefit (red curve) per patient for predicting the risk of delayed extubation. Within the threshold risk range of 0%∼30%, intervention decisions based on the predictive model are clearly beneficial (Figure 5).

### External validation

The pre- and intraoperative characteristics in the validation cohort were described in Table 1. The prediction model indicated good discrimination in estimating the risk of delayed extubation, with a C-statistic value of 0.785 in validation cohort. The sensitivity and specificity based on AUROC curve were 89.5% and 62.0%, respectively (Figure 3(B)). Calibration plot with H-L type χ^2^ statistic value of 0.113 also showed good calibration in validation cohort (Supplementary Figure 2).

## Discussion

The incidence of delayed extubation after thoracoscopic lung cancer surgery was 1.52%. This study identified eight independent predictors for delayed extubation. Using these eight candidates to construct a novel nomogram to estimate the decision to delayed extubation, with good C-statistic and calibration both in internal and external validation. The DCA indicated the clinical usefulness of the nomogram, suggesting that intervention decisions based on the predictive model were clearly beneficial when the threshold risk range of 0%∼30%.

Previous studies have identified some underlying predictors for delayed extubation [9,13–18], including age, ASA grade, body mass index (BMI), baseline lung function, operative time, case end time, extent of resection, volume of crystalloid and transfusion. By comparison, this study also demonstrated that age, BMI, FEV_1_/FVC, lymph nodes calcification, anaesthesia type, intraoperative transfusion, prolonged operative time and operation later than 6 p.m. were independent predictors for delayed extubation. Among these factors, lymph node calcification and anaesthesia type were rarely reported in the literature, and the latter may be an important adjustable factor to reduce the incidence of delayed extubation.

The higher rate of delayed extubation due to lymph node calcification may be possibly explained by the increased difficulty of tissue separation and dissection when thoracoscopic procedure is applied [23], as well as the associated prolonged operative time and increased intraoperative complications [24]. An important finding of this study was that preoperative application of TPVB, administered with 15 mL 0.5% ropivacaine through T4-T5 under ultrasound guidance, could reduce the risk of delayed extubation. The probably reason may be due to reduced intraoperative opioid dosage and improved postoperative pain control [25,26], thereby increasing patient tolerance to tracheal and chest tube [27]. Similar to our findings, epidural analgesia usage has been established to reduce the odds of mechanical ventilation and associated delayed extubation after thoracotomy [15,28].

Moreover, the combination of the predictive ability of operation later than 6 P.M. as well as the lack of predictive ability of attending handoff implicated that the timing of the operation weighs more than attending handoff and may be a direct cause for delayed extubation. This is consistent with purported statements that the timing of the operation, rather than attending handoff, has an impact on postoperative complications [9,29,30]. Adversely, Anastasian et al. reviewed the data of 37,824 patients who underwent general anaesthesia with an endotracheal tube for surgery and concluded that attending handoff was an independent risk factor for delayed extubation [19]. The differences among the two studies lie in the inclusion of the population and the fact that this study did not categorize the specific time of attending handoff.

For model construction, this study was the first paper to predict delayed extubation, and all variables inserted in the predictive model were quantifiable predictors readily available to the thoracic clinicians. Besides, the nomogram can provide a visual point system to estimate the probability of delayed extubation with good discriminations after internal and external validation. Bias-corrected calibration curve showed that the model could accurately predict the occurrence of delayed extubation when the observed probability of delayed extubation was less than 15%. In the external validation cohort, this prediction model showed good calibration. Also, the depicted DCA showed that, within the threshold risk range of 0%∼30%, intervention decisions based on the predictive model are clearly beneficial.

Several limitations are among our research. First, as a monocentric retrospective study based on prospectively collected data, it has the inherent design biases. Second, although the prediction model demonstrated good efficacy in both internal and external validation in this study, external validation in a multicentre setting was still needed. Third, whether the four adjustable predictors identified in this study, especially the usage of TPVB, can reduce the occurrence of delayed extubation after surgery still needs to be verified by prospective randomized trials.

## Conclusions

By reviewing 12,392 patients who underwent thoracoscopic lung cancer surgery, this cohort study identified eight independent predictors for delayed extubation, among which lymph node calcification and anaesthesia type were not commonly reported. Using these eight candidates, we constructed a novel nomogram, which can reliably identify patients at high risk for the decision to delayed extubation, with good performance in both internal and external validation. Optimizing four modifiable factors including BMI, FEV_1_/FVC, TPVB usage and operation later than 6 p.m. may reduce the risk of delayed extubation.

## Supplementary Material

Supplemental Material

## Data Availability

Our research team could provide original data under reasonable request and with permission from Shanghai Chest Hospital.
