# Supplementary material for: A novel nomogram for predicting the decision to delayed extubation after thoracoscopic lung cancer surgery
Source: Ann Med. 2023 Mar 4;55(1):800–7. doi: 10.1080/07853890.2022.2160490 (PMC9987746; doi:10.1080/07853890.2022.2160490)
Supplement: Supplemental Material [file IANN_A_2160490_SM2949.docx]

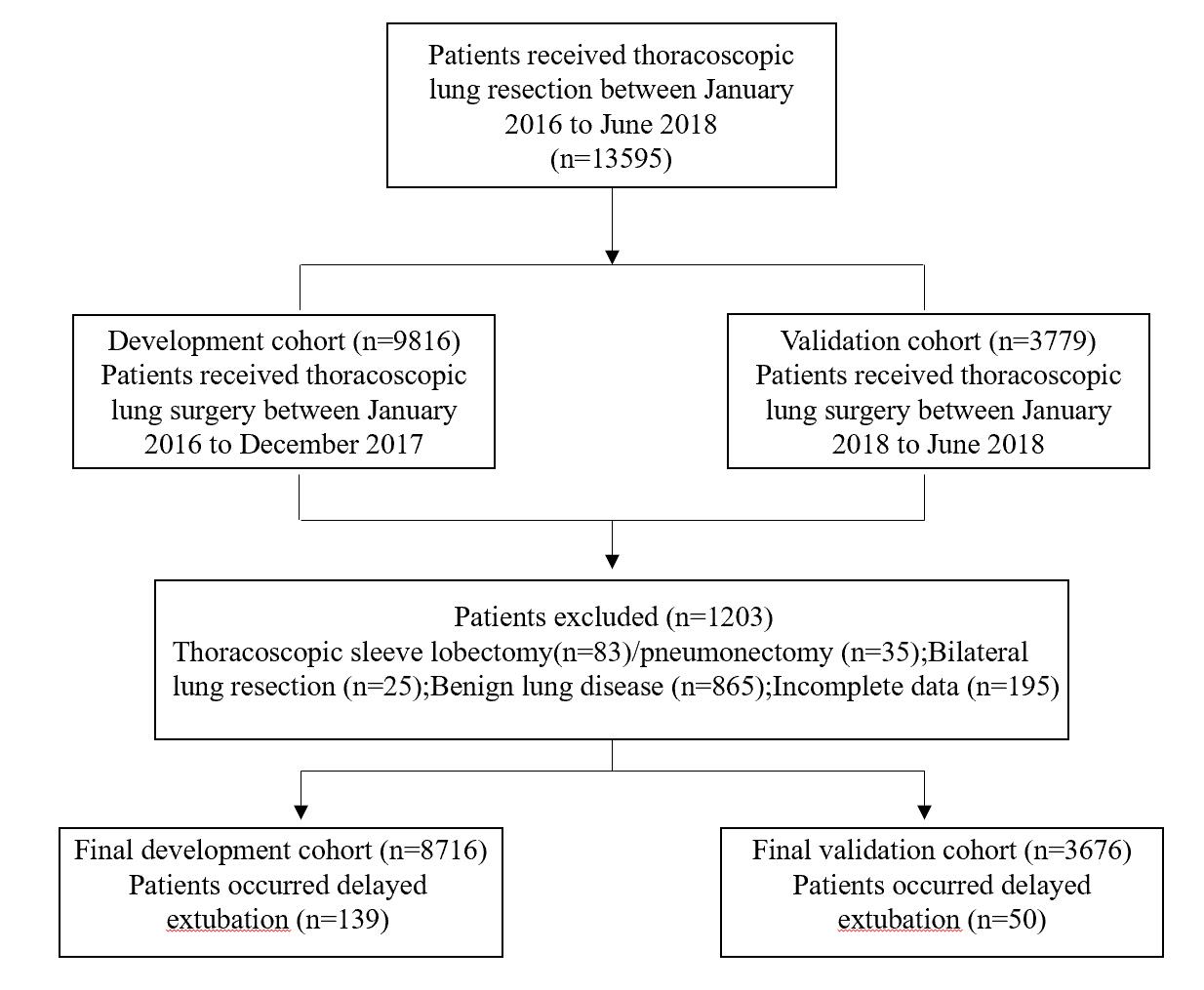


**Supplementary Figure 1** **Patient flowchart.**


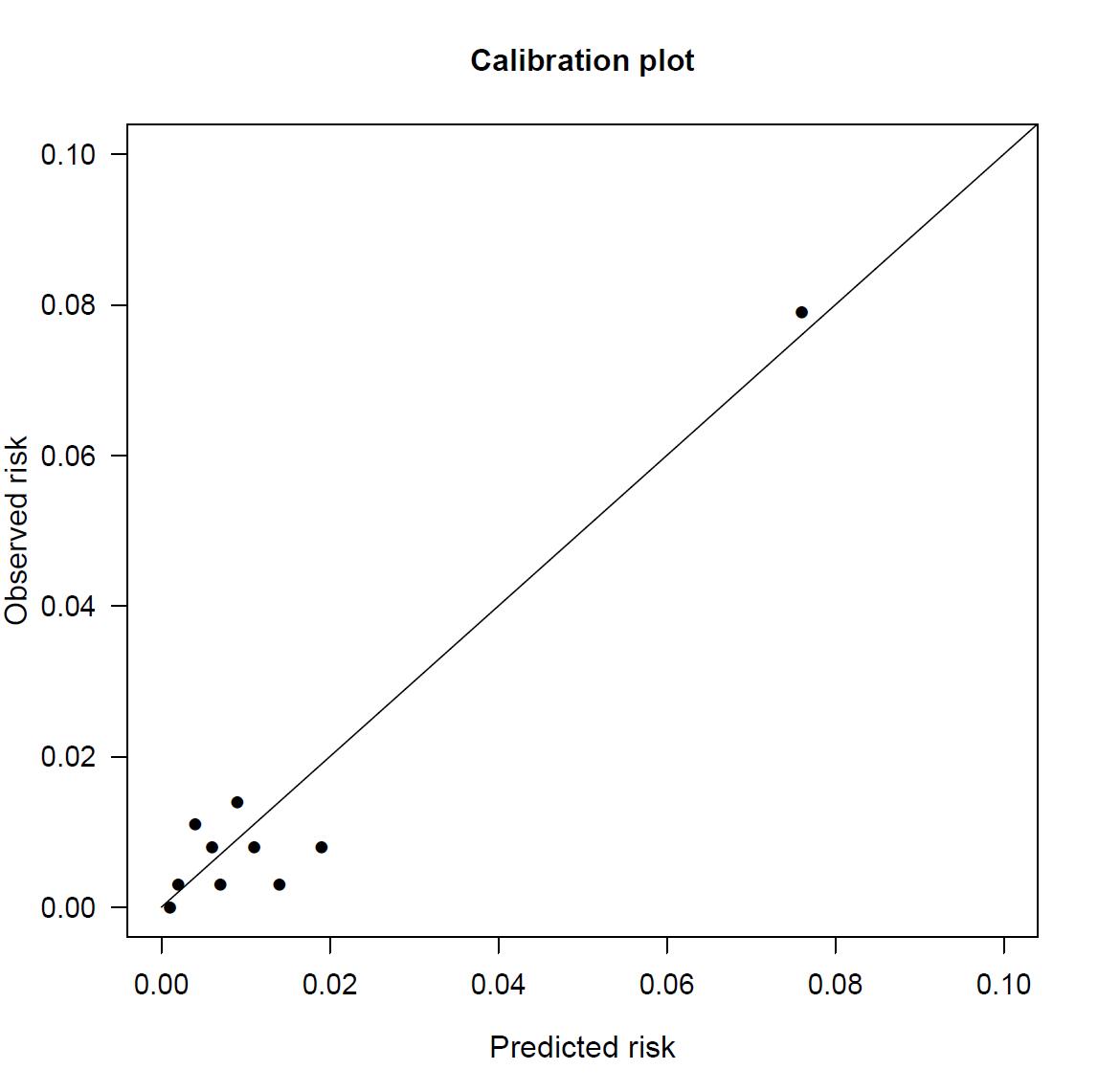


**Supplementary Figure 2 External calibration plot.**

F=12.963, df=8. P=0.113. Calibration plot of mean observed probability of delayed extubation (y-axis) vs. predicted probability (x-axis), in both development and external validation cohort.
